# Quality of medicines: Deficiencies found by Brazilian Health Regulatory Agency (ANVISA) on good manufacturing practices international inspections

**DOI:** 10.1371/journal.pone.0202084

**Published:** 2018-08-08

**Authors:** Andrea Renata Cornelio Geyer, Varley Dias Sousa, Dâmaris Silveira

**Affiliations:** 1 Brazilian Health Regulatory Agency (ANVISA), Brasilia, DF, Brazil; 2 Department of Pharmaceutical Sciences, Universidade de Brasilia (UnB), Brasilia, DF, Brazil; Robert Gordon University, UNITED KINGDOM

## Abstract

The circulation of poor quality medicines, especially in the developing countries, is a public health concern. Compliance with good manufacturing practices (GMP) is essential to ensure the quality, efficacy, and safety of medicines. This study evaluated the outcomes of the Brazilian Health Regulatory Agency’s (ANVISA) international inspections of two years (2015 and 2016) and compared these to those of other regulatory authorities. The information from 255 inspection reports was analyzed, and the type and extent of deficiencies were collected. In the period evaluated, 62.75% of ANVISA-inspected companies were classified as GMP “satisfactory,” 24.71% were classified as having “on demand” status, and 12.55% of inspections concluded that the company did not comply with Brazilian GMP regulations (“unsatisfactory”). The most common areas of deficiency were documentation (28.63%) and premises (26.27%). The pattern of deficiencies was similar to the findings of other regulatory agencies. However, ANVISA detected a more significant number of non-compliance results than other authorities, which may be caused by differences in classifications adopted by each Agency. Furthermore, manufacturers inspected by ANVISA may follow different standards and practices for products manufactured for the Brazilian market. Disclosure of main GMP deficiencies found can be useful for encouraging the industry to comply with GMP, and additional guidelines in the specific areas where deficiencies are often identified may be useful to industry to improve GMP compliance. Harmonization of GMP guidelines and inspection procedures are the key steps to avoid duplicate work, but regulatory authorities also need to work together to enforce the proper level of GMP compliance by pharmaceutical manufacturers, assuring high quality and safe medicines supply.

## Introduction

Good manufacturing practices (GMP) is the part of quality management which ensures that products are produced and controlled in conformance with quality standards appropriate to their intended use and as required by the marketing authorization (MA)[1–4]. Adherence to the GMP regulations contributes to reaching key quality attributes, including and are not limited to identity, strength, quality, and purity of drug products [5].

The primary objective of GMP is to manage and minimize the inherent risks in pharmaceutical manufacture to guarantee the quality, safety, and efficacy of products [1–3], assuring the highest standards of efficacy, quality, and safety in any process that involves the manufacture of health products [6].

The spread of poor quality (substandard, spurious, falsely labeled, falsified, or counterfeit) medicines, especially in developing countries, has been a global public health concern [7–12]. Protecting the public from exposure to poor quality medicines requires the presence of a robust medicines quality assurance system [8]. Poor compliance with GMP standards can lead to substandard medicine production, lack of sterility, and product mix-ups, which may happen accidentally (such as through human error) or as a result of insufficient resources (e.g., expertise, appropriate manufacturing infrastructure, or human and financial resources). Reliable data on the prevalence of substandard medicines are lacking, but emerging economies and developing countries are likely to face significant problems with substandard medications because of poor drug regulation and poor pharmaceutical industry compliance with GMP [9,10].

A keystone of effective medicine regulation is ensuring that the medicines available within a market meet appropriate quality standards [12]. On the other hand, currently, regulatory activities are particularly onerous and complex, due to the new technologies and the globalization of pharmaceutical activities [12,13]. Therefore, organizations, institutions, regulatory authorities, and the pharmaceutical industry are seeking maximum harmonization of GMP guidelines to avoid the duplication of regulatory efforts [6].

GMP inspections are critical activities that can have a devastating impact on a company’s profitability and may result in severe regulatory consequences if violations are uncovered, including recalls, lost sales, shutting down of lines or entire facilities and a negative impact on their reputation [14,15]. Furthermore, poor compliance with GMP guidelines may lead to shortages of essential pharmaceuticals products, that can potentially have major effects on the quality of medical care, including medication errors, treatment delays, adverse outcomes, and increased health care costs [16–21].

In Brazil, the health regulatory agency (HRA) responsible for pharmaceutical products regulation is the Brazilian Health Regulatory Agency (ANVISA), created by a Federal Law in 1999. ANVISA’s primary goal is to exercise health surveillance over goods and services, including processes, ingredients, and technologies that pose any health risks [22]. In the same year of its creation, ANVISA issued a regulation requiring international inspections to issue a GMP certificate for imported medicines manufacturers. The GMP certificate must be presented for MA and related variations in Brazil [23].

Presently, the GMP certificate in Brazil is valid for two years. Renewal may be requested for ANVISA, which will decide if another inspection will be needed based on a risk assessment, considering the GMP compliance history, time elapsed since the last inspection, new products or line inclusions, marketing complaints, and product quality review information [24].

It is important to note that regulatory transparency policies are essential for strengthening HRA, establishing society confidence in health authority work, supporting the convergence of policies and procedures across agencies, avoiding duplication of efforts, and reducing costs and workloads. Moreover, regulatory transparency policies may enable HRA with limited capacity and resources to operate more efficiently, strengthening the public health system [13]. Public disclosure of the main deficiencies found on inspections is essential for regulatory transparency.

The primary objective of the present study was to evaluate the outcomes of ANVISA foreign inspections in the preceding two years (2015 and 2016), conducting a review of found deficiencies. Disclosure of the leading found GMP deficiencies can be useful for encouraging the industry to comply with GMP, sharing of experiences between international regulatory authorities, improving legislation requirements and increasing availability of good quality medicines.

## Materials and methods

The information contained in the inspection reports was analyzed, and the type and extent of deficiencies were collected in a form created using the system FormSUS. The study covered inspections performed across a 2-year period, the time that a GMP certificate is valid in Brazil, from 1 January 2015 to 31 December 2016.

Results were collected from a total of 255 inspection reports. An Excel database was created with all the collected issues, and these were classified in four ways.

First, the conclusion of the inspection was grouped by company compliance status according to the national standard operating procedure (SOP) for company classification (POP-O-SNVS-014) [25], as “satisfactory,” “on demand” or “unsatisfactory.” The results were also grouped by country, production lines and if the company was previously inspected or not.

Second, for companies where deficiencies were found, the number and seriousness of these were collected (critical, major, and minor). The means, medians, minimums, maximums (range) and standard deviations of each deficiency were also calculated.

Third, deficiencies were grouped per the titles and chapters of GMP regulations in Brazil.

Finally, deficiencies found more frequently were listed descriptively regarding the article (item) of the regulation and were then quantified. Their frequency was also quantified by the total number of deficiencies.

## Results

**Fig 1:**
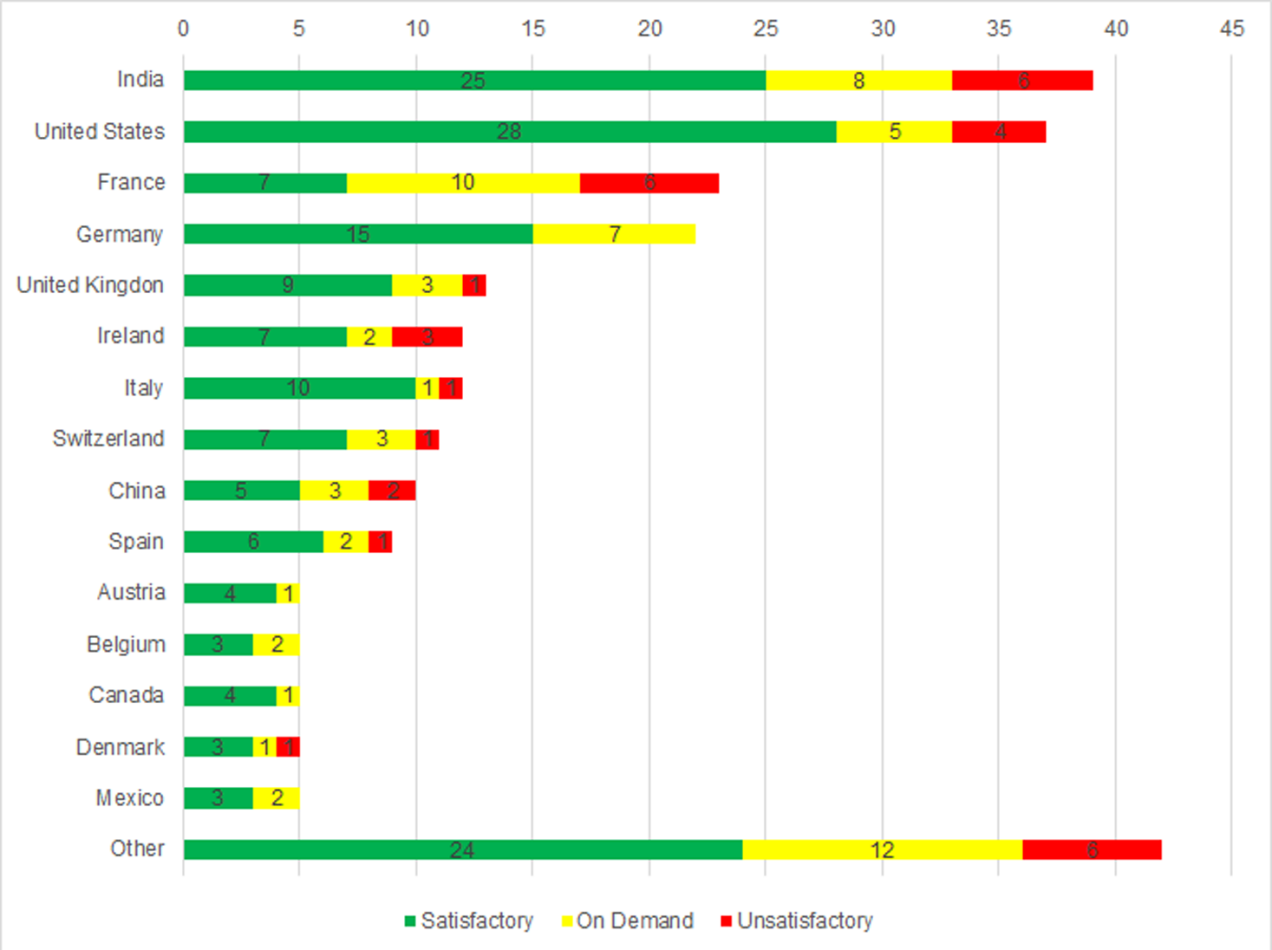
ANVISA inspections per country and the respective company classification.

Between January 1, 2015, and December 31, 2016, ANVISA performed 255 international inspections of drug products, in 41 countries. Each country’s inspections, outcomes and the respective percentage of each conclusion category are described in Table 1. Fig 1 shows the absolute number of each result per country. Countries where fewer than five inspections were performed (Poland, Sweden, Japan, Netherlands, Singapore, Colombia, South Korea, Israel, Paraguay, Ukraine, Australia, Costa Rica, Croatia, Ecuador, Slovenia, Philippines, Finland, Greece, Latvia, Malaysia, Portugal, Romania, Russia, Thailand, Czech Republic, and Turkey), are grouped as other.

**Table 1:** Number of inspections per country and conclusions. Countries of which the number of inspections was fewer than five are grouped as “other.”

	Number of Inspections	Percentage	Satisfactory (%)	On Demand (%)	Unsatisfactory (%)
India	39	15.29%	25 (64.10%)	8 (20.51%)	6 (15.38%)
United States	37	14.51%	28 (75.68%)	5 (13.51%)	4 (10.81%)
France	23	9.02%	7 (30.43%)	10 (43.48%)	6 (26.09%)
Germany	22	8.63%	15 (68.18%)	7 (31.82%)	-
United Kingdom	13	5.10%	9 (69.23%)	3 (23.08%)	1 (7.69%)
Ireland	12	4.71%	7 (58.33%)	2 (16.67%)	3 (25%)
Italy	12	4.71%	10 (83.33%)	1 (8.33%)	1 (8.33%)
Switzerland	11	4.31%	7 (63.64%)	3 (27.27%)	1 (9.09%)
China	10	3.92%	5 (50%)	3 (30%)	2 (20%)
Spain	9	3.53%	6 (66.67%)	2 (22.22%)	1 (11.11%)
Austria	5	1.96%	4 (80%)	1 (20%)	-
Belgium	5	1.96%	3 (60%)	2 (40%)	-
Canada	5	1.96%	4 (80%)	1 (20%)	-
Denmark	5	1.96%	3 (60%)	1 (20%)	1 (20%)
Mexico	5	1.96%	3 (60%)	2 (40%)	-
Other	42	16.47%	24 (57.14%)	12 (28.57%)	6 (14.29%)
Total	255	100%	160 (62.75%)	63 (24.71%)	32 (12.55%)

**Fig 2:**
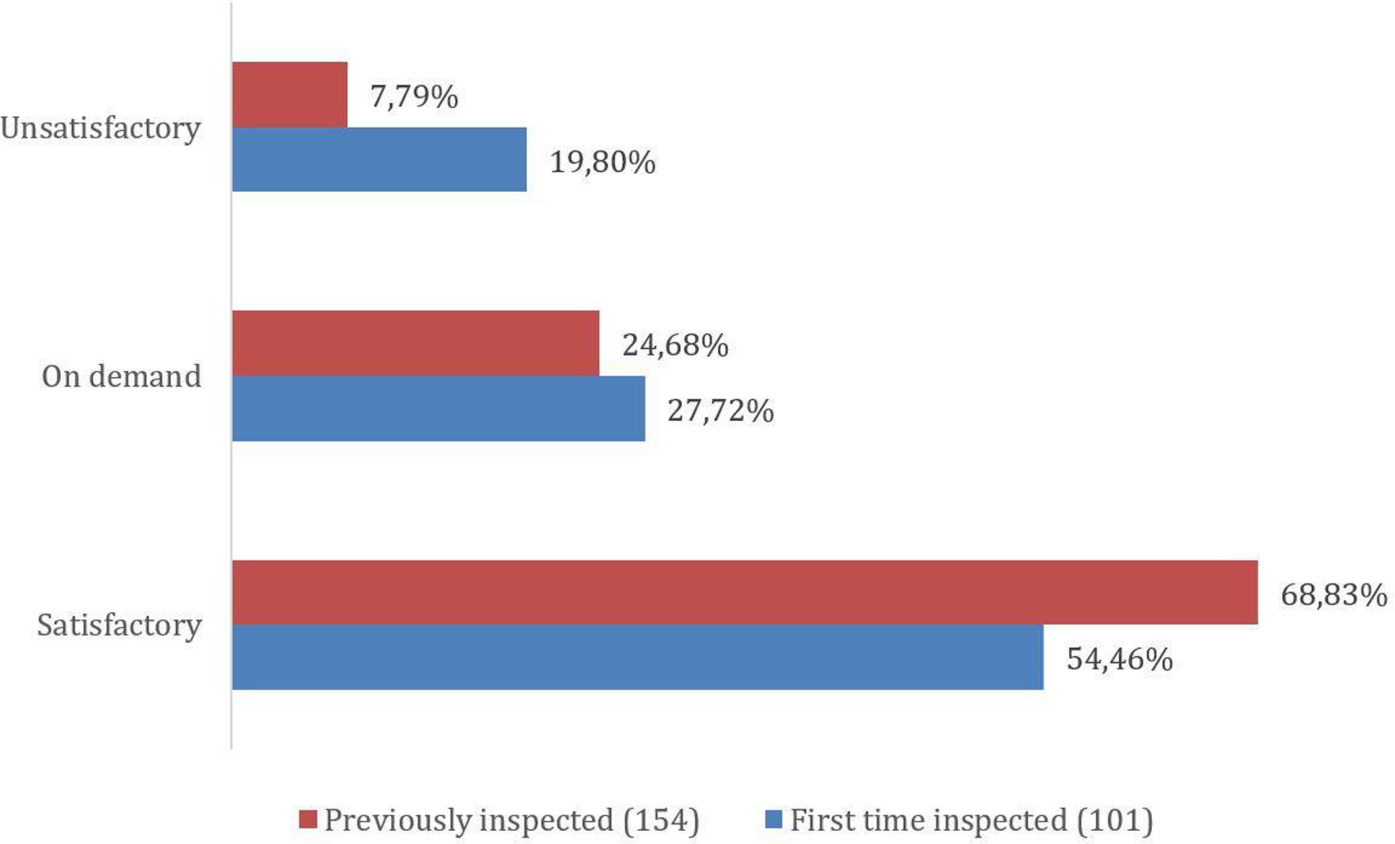
Outcomes of companies first time inspected and companies that had been previously inspected.

Of the total of 255 inspections, 101 (39.61%) of the companies were being inspected for the first time, while 154 (60.39%) had been inspected before. The outcomes found for each situation are presented in Fig 2.

Sterile products were covered in 125 inspections (49.02%), 131 referred to non-sterile solids (51.37%), 25 to non-sterile liquids (9.80%), and 10 to non-sterile semi-solids (3.92%). One inspection may cover more than one production line, and the conclusion may be different for each line. We identified four situations where it occurred, for two companies in India, one in the United States and one in the United Kingdom. Three of those companies were classified as unsatisfactory for sterile production line and as satisfactory or as on demand for nonsterile pharmaceutical dosages forms. The result described in Table 1 was counted just once, as unsatisfactory since another inspection would be required. Conclusions by each production line are described in Table 2.

**Table 2:** Number of inspections per production line and conclusions.

	Number of Inspections	Satisfactory	On Demand	Unsatisfactory
Sterile products	125	75 (60.00%)	34 (27.20%)	16 (12.80%)
Nonsterile solids	131	93 (70.99%)	26 (19.85%)	12 (9.16%)
Nonsterile liquids	25	13 (52.00%)	9 (36.00%)	3 (12.00%)
Nonsterile semisolids	10	3 (30.00%)	5 (50.00%)	2 (20.00%)

**Fig 3:**
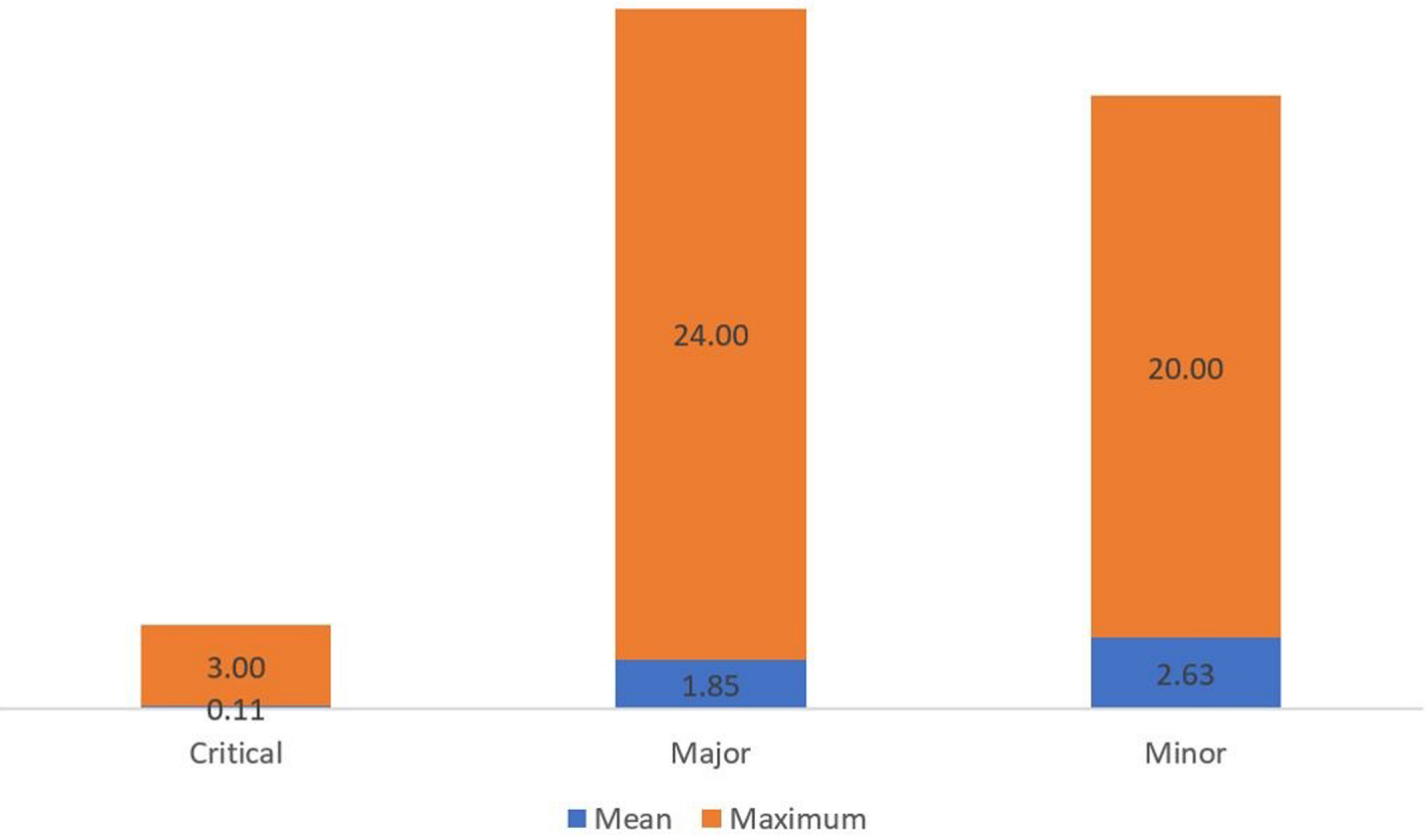
Mean and maximum number of each kind of deficiency found per ANVISA inspections.

**Fig 4:**
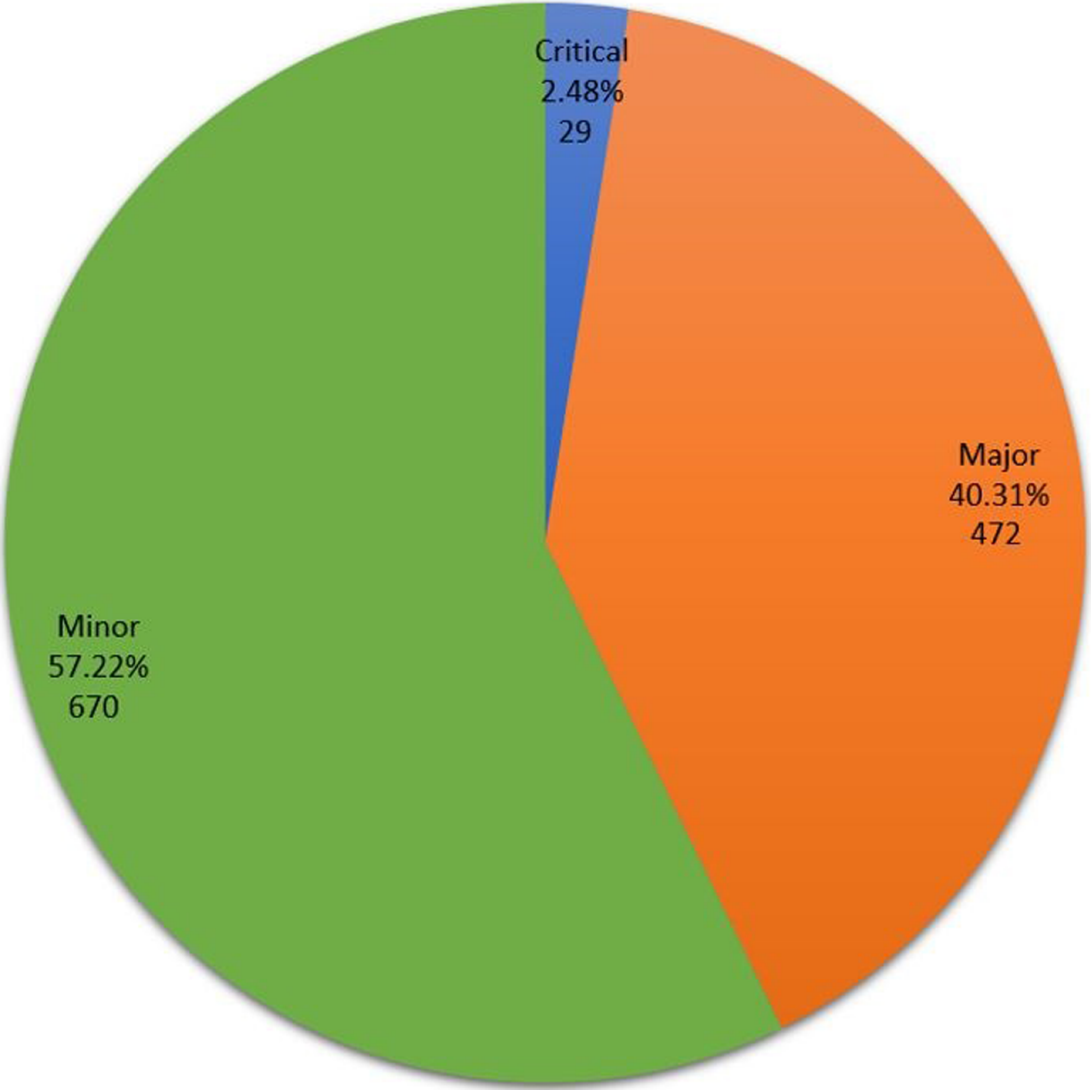
Distribution of criticality of deficiencies found during ANVISA inspections.

A total of 1171 GMP deficiencies were found in these inspections. The mean number was 4.59 per inspection (*SD* = 6.2), and the median was 2 (range: 0–42 deficiencies). Critical deficiencies were found in 19 inspections (7.45%), major deficiencies were observed in 111 (43.53%) inspections, and minor nonconformities were found in 165 (64.71%) inspections. Fig 3 represents the mean and maximum of each kind of deficiency found. A total number of 29 critical (2.48%), 472 major (40.31%), and 670 minor (57.22%) deficiencies were found, as shown in Fig 4.

**Fig 5:**
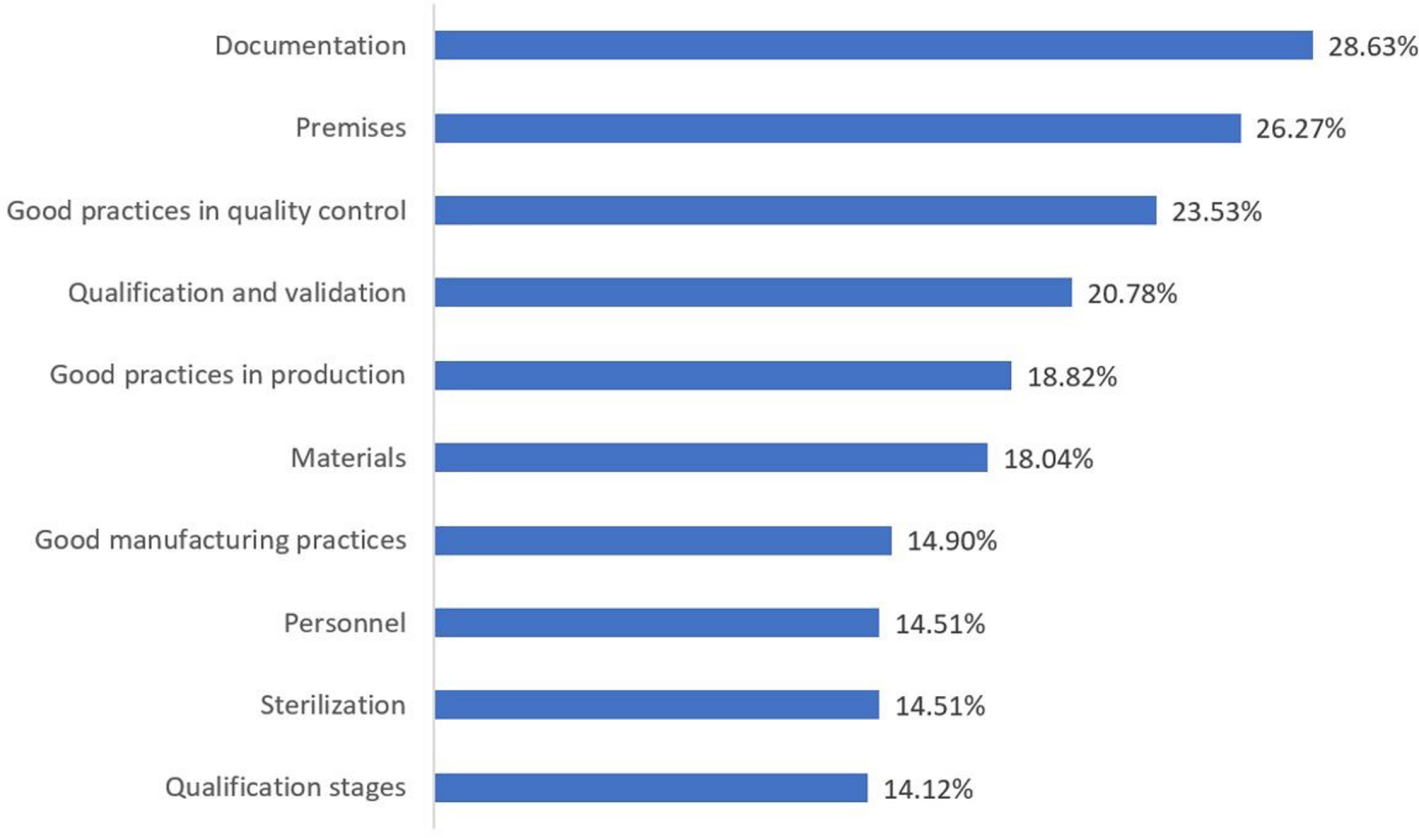
Top 10 areas in which deficiencies were found during ANVISA inspections (as a percentage of inspected companies).

Table 3 shows the deficiencies found per title and chapter of the Brazilian regulations and their international reference. The top 10 most common areas with deficiencies are presented in Fig 5.

**Table 3:** Brazilian regulation titles and chapters, international references, and frequency of deficiencies found during ANVISA inspections.

Title/Chapter	Inspections with deficiency found (% of inspections)	Number of Deficiencies (% of total deficiencies)
**Title II–Quality management in the drug industry: philosophy and essential elements**	**Reference: WHO Technical Report Series 908, 2003—Annex 4 [26]**	
Introduction (Art. 6–9)	3 (1.18%)	4 (0.35%)
Chapter I–Quality assurance (Art. 10–12)	23 (9.02%)	26 (2.25%)
Chapter II–Good manufacturing practices for pharmaceutical products (Art. 13)	38 (14.90%)	38 (3.30%)
Chapter III–Sanitation and hygiene (Art. 14)	7 (2.75%)	7 (0.61%)
Chapter IV–Qualification and validation (Art. 15–25)	53 (20.78%)	73 (6.33%)
Chapter V–Complaints (Art. 26–34)	8 (3.14%)	9 (0.78%)
Chapter VI–Product recalls (Art. 35–42)	3 (1.18%)	3 (0.26%)
Chapter VII–Contract production and analysis (Art. 43–60)	2 (0.78%)	2 (0.17%)
Chapter VIII–Self-inspection and quality audits (Art. 61–69)	17 (6.67%)	24 (2.08%)
Chapter IX–Personnel (Art. 70–85)	37 (14.51%)	49 (4.25%)
Chapter X–Training (Art. 86–91)	6 (2.35%)	6 (0.52%)
Chapter XI–Personal hygiene (Art. 92–101)	2 (0.78%)	2 (0.17%)
Chapter XII–Premises (Art. 102–138)	67 (26.27%)	138 (11.97%)
Chapter XIII–Equipment (Art. 139–152)	27 (10.59%)	32 (2.78%)
Chapter XIV–Materials (Art. 153–196)	46 (18.04%)	59 (5.12%)
Chapter XV–Documentation (Art. 197–244)	73 (28.63%)	117 (10.15%)
Chapter XVI–Good practices in production (Art. 245–280)	48 (18.82%)	86 (7.46%)
Chapter XVII–Good practices in quality control (Art. 281–305)	60 (23.53%)	94 (8.15%)
**Title III–Sterile Products**	**Reference: WHO Technical Report Series 902, 2002—Annex 6 [27]**	
Chapter I–General considerations (Art. 306–309)	1 (0.39%)	1 (0.09%)
Chapter II–Quality control (Art. 310–314)	12 (4.71%)	14 (1.21%)
Chapter III–Sanitation (Art. 315–318)	13 (5.10%)	16 (1.39%)
Chapter IV–Manufacture of sterile preparations (Art. 319–347)	17 (6.67%)	23 (1.99%)
Chapter V–Sterilization (Art. 348–427)	37 (14.51%)	65 (5.64%)
**Title IV–Biological Products**	**Reference: WHO Technical Report Series 822, 1992—Annex 1[28]**	
Chapter I–Scope (Art. 428–430)		
Chapter II–General considerations (Art. 431–434)		
Chapter III–Personnel (Art. 435–439)		
Chapter IV–Premises and equipment (Art. 440–451)	2 (0.78%)	2 (0.17%)
Chapter V–Animal quarters (Art. 452–460)		
**Title V–Validation**	**Reference: WHO Technical Report Series 937, 2006—Annex 4 [29]**	
Chapter I–Introduction (Art. 461)	20 (7.84%)	20 (1.73%)
Chapter II–Relationship between validation and qualification (Art. 462)		
Chapter III–Validation (Art. 463–474)	23 (9.02%)	29 (2.52%)
Chapter IV–Qualification (Art. 475–477)	18 (7.06%)	23 (1.99%)
Chapter V–Calibration and verification (Art. 478–483)	4 (1.57%)	9 (0.78%)
Chapter VI–Validation master plan (Art. 484)	5 (1.96%)	5 (0.43%)
Chapter VII—Qualification and validation protocols (Art. 485–488)	4 (1.57%)	5 (0.43%)
Chapter VIII–Qualification and validation reports (Art. 489–495)	7 (2.75%)	9 (0.78%)
Chapter IX–Qualification stages (Art. 496–521)	36 (14.12%)	48 (4.16%)
Chapter X–Change control (Art. 522–524)	10 (3.92%)	16 (1.39%)
Chapter XI–Personnel (Art. 525–526)		
**Title VI–Water for Pharmaceutical Use**	**Reference: WHO Technical Report Series 929, 2005—Annex 3 [30]**	
Chapter I–Requirements for pharmaceutical water systems (Art. 527–531)	3 (1.18%)	3 (0.26%)
Chapter II–Water quality specifications (Art. 532–538)	3 (1.18%)	4 (0.35%)
Chapter III–Water purification methods (Art. 539–549)	8 (3.14%)	8 (0.69%)
Chapter IV–Water purification, storage, and distribution systems (Art. 550–560)	18 (7.06%)	22 (1.91%)
Chapter V–Operational considerations (Art. 561–567)	14 (5.49%)	19 (1.65%)
Chapter VI–Maintenance of water systems (Art. 568)	1 (0.39%)	1 (0.09%)
Chapter VII–System reviews (Art. 569)	22 (8.63%)	22 (1.91%)
**Title VII–Computerized Systems (Art. 570–590)**	**Reference: EMA GMP Guide (Annex 11) and PIC/S GMP Guide [31,32]**	
	15 (5.88%)	22 (1.91%)
**Title VIII–Good Manufacturing Practices for the manufacture of herbal medicines**	**Reference: WHO Technical Report Series 937, 2006—Annex 3 [33]**	
Chapter I–General considerations (Art. 591–592)		
Chapter II–Quality assurance (Art. 593)		
Chapter III–Sanitation and hygiene (Art. 594)		
Chapter IV–Validation (Art. 595)	3 (1.18%)	3 (0.26%)
Chapter V–Self-inspection (Art. 596)		
Chapter VI–Personnel (Art. 597)		
Chapter VII–Training (Art. 598)	1 (0.39%)	1 (0.09%)
Chapter VIII–Personal hygiene (Art. 599)		
Chapter IX–Equipment (Art. 600)	1 (0.39%)	1 (0.09%)
Chapter X–Reference samples and standards (Art. 601–602)		
Chapter XI–Documentation (Art. 603–606)		
Chapter XII–Quality Control (Art. 607)		

The deficiencies found more frequently, the correspondent article (item) of the Brazilian regulation and the international reference are shown in Table 4. All articles cited more than ten times in the period are listed.

**Table 4:** Deficiencies often found during ANVISA inspections (article of Brazilian regulation), international correspondence, and frequency of inspections in which the specific deficiencies were found.

Deficiency (Article)	International correspondence	Frequency (% of inspections found)
Products are not consistently produced and controlled to the quality standards appropriate to their intended use and as required by the marketing authorization (Art. 13).	2.1 of WHO Technical Report Series 908, 2003—Annex 4	38 (14.90%)
Water systems (purified water and water for injection) not reviewed at appropriate regular intervals or not all the relevant topics considered (Art. 569).	7.5 of WHO Technical Report Series 929, 2005—Annex 3	22 (8.63%)
Documentation does not cover all aspects of GMP, for example does not ensure that authorized person have all the information necessary to decide to release a batch of a drug for sale, does not provide traceability that will permit investigation or the availability of the data needed for validation, review, and statistical analysis (Art. 197).	15.1 of WHO Technical Report Series 908, 2003—Annex 4	21 (8.24%)
Time limits for storage of equipment before and after cleaning not stated or based on data validation (Art. 263).	16.18 of WHO Technical Report Series 908, 2003—Annex 4	21 (8.24%)
Qualification and validation considered as one-off exercises. There is no on-going monitoring program based on a periodic review (Art. 19).	4.5 of WHO Technical Report Series 908, 2003—Annex 4	20 (7.84%)
Quality assurance system is inappropriate for the manufacturing of pharmaceutical products. For example, does not ensure that deviations are reported, investigated, and recorded (Art. 11).	1.2 of WHO Technical Report Series 908, 2003—Annex 4	19 (7.45%)
Storage areas not designed or adapted to ensure good storage conditions, e.g., temperature and humidity not provided, controlled, monitored, and recorded where appropriate (Art. 117).	12.16 of WHO Technical Report Series 908, 2003—Annex 4	19 (7.45%)
Validation of processes and systems does not establish confidence that the manufactured products will consistently meet their product specifications. (Art. 461)	1 of WHO Technical Report Series 937, 2006—Annex 4	19 (7.45%)
Analytical test methods, automated systems, or cleaning procedures not validated (Art. 25).	4.11 of WHO Technical Report Series 908, 2003—Annex 4	18 (7.06%)
Quality control does not properly evaluate the quality and stability of finished pharmaceutical products or, when necessary, of starting materials and intermediate products (Art. 302).	17.23 of WHO Technical Report Series 908, 2003—Annex 4	16 (6.27%)
Equipment, utility, or system not maintained, monitored, and calibrated according to a regular schedule (Art. 476).	6.2 of WHO Technical Report Series 937, 2006—Annex 4	16 (6.27%)
Sampling not being conducted in such a way as to prevent contamination or cross-contamination (Art. 123).	12.22 of WHO Technical Report Series 908, 2003—Annex 4	13 (5.10%)
Documents not regularly reviewed or kept up to date (Art. 201).	15.5 of WHO Technical Report Series 908, 2003—Annex 4	13 (5.10%)
The quality control department does not have adequate resources to ensure that all the quality control arrangements are effectively and reliably carried out. (Art. 283).	17.3 of WHO Technical Report Series 908, 2003—Annex 4	13 (5.10%)
Identity test not conducted on a sample from each container of starting material (Art. 294).	17.15 of WHO Technical Report Series 908, 2003—Annex 4	13 (5.10%)
There is no documentary evidence of design qualification, installation qualification, operational qualification, performance qualification, or process validation (Art. 17).	4.3 of WHO Technical Report Series 908, 2003—Annex 4	11 (4.31%)
The heads of the production and quality control and quality assurance do not share, or jointly exercise, responsibilities relating to quality, such as monitoring and control of the manufacturing environment, process validation and calibration of analytical apparatus, approval and monitoring of suppliers of materials, or approval and monitoring of contract manufacturers (Art. 77).	9.8 of WHO Technical Report Series 908, 2003—Annex 4	11 (4.31%)
Production areas do not have air treatment systems appropriate for the products manufactured, operations made, and external environment (including air filtration) to prevent contamination and cross-contamination, as well as control of temperature and, where necessary, humidity and differential pressures (Art. 132).	12.30 of WHO Technical Report Series 908, 2003—Annex 4	11 (4.31%)
The documents present ambiguous contents (Art. 200).	15.4 of WHO Technical Report Series 908, 2003—Annex 4	11 (4.31%)
Premises are not located, designed, constructed, adapted, or maintained to suit the operations to be carried out (Art. 102).	12.1 of WHO Technical Report Series 908, 2003—Annex 4	10 (3.92%)
Where dust is generated, e.g., during sampling, weighing, mixing and processing operations, and packaging of powder, measures are not taken to avoid cross-contamination and facilitate cleaning (Art. 104).	12.3 of WHO Technical Report Series 908, 2003—Annex 4	10 (3.92%)

## Discussion

According to the Brazilian SOP (POP-O-SNVS-014) [25], deficiencies are classified as critical, major, and minor. A critical deficiency is a deficiency that probably results in a product that does not comply with essential attributes of the marketing authorization or can present an immediate or latent health risk. Additionally, any deficiency involving fraud (product or data falsification) or tampering is categorized as critical. A deficiency that can result in a product not compatible with the key attributes of the marketing authorization is considered as major. If the deficiency cannot be classified as either critical or major, but is a deviation from the GMP, it is classified as minor. The procedure also states that if during the inspection, up to five minor deficiencies are observed, the company is classified as satisfactory. If the inspectors found six or more minor deficiencies and/or less than five major deficiencies, it is classified as on demand, which means that the company has 120 calendar days to comply with all pending requirements described in the report and to present documented evidence to ANVISA for GMP certificate issuance [24]. If one or more critical deficiencies and/or six or more major deficiencies are observed, the company is classified as unsatisfactory. In this case, the GMP certificate application is rejected, the foreign company must be inspected again for GMP certificate issuance, and the requesting company in Brazil (the importing company) needs to reapply for the certificate, paying the correspondent fee, causing a delay in the MA process. If the product is already registered in Brazil, some penalties may be applied, like importation preclusion or product recall.

In the period under analysis, 62.75% of ANVISA-inspected companies were classified as satisfactory, 24.71% received on demand status, and 12.55% of inspections concluded that the company did not comply with the GMP (unsatisfactory). As presented in Fig 4, most deficiencies found were considered minor (57.22%), however many major deficiencies (40.31%) were also found. Critical deficiencies were a small part of the total (2.48%).

The United States of America Food and Drug Administration (U.S. FDA) classifies the conclusions of inspections as official action indicated (OAI), when significant unsuitable conditions or practices are found, and regulatory action is warranted to address the establishment’s lack of compliance with the statute(s) or regulation(s); voluntary action indicated (VAI), when objectionable conditions or practices that do not meet the threshold of regulatory significance are found; and no action indicated (NAI), when no objectionable conditions or practices are found during the inspection, or the significance of the documented objectionable conditions found does not justify further action [34]. Inspection classifications can be searched on the inspections database, available on the U.S. FDA website [35]. A search on the database for the fiscal year 2016 (inspections between October 2015 and September 2016) with the entry “Project 56—Drug Quality Assurance,” resulted in 1,429 records (824 from the U.S.). Of the total, 582 (40.73%) were classified as NAI, 807 (56.47%) as VAI, and 40 (2.80%) as OAI.

According to the European Medicines Agency (EMA) 2016 annual report, approximately 1% of the inspections conducted by European Union authorities led to a non-compliance statement (24 out of 2,293). The issued non-compliance statements referred mostly to the Indian (11.11%), Chinese (6.77%), and U.S. (3.37%) manufacturing plants inspected in 2016 [36].

Health Canada reports the inspection work every year in an annual inspection summary report. In the last one published, for the 2014–2015 fiscal year (from April 1, 2014, to March 31, 2015), the Canadian inspectorate conducted 442 domestic drug GMP inspections, 227 in drug manufacturers, and made 3,096 observations. Of these observations, 0.5% (17 issues) were categorized as Risk 1 (critical), 50.2% (1,612) as Risk 2 (major), and 49.3% (1,579) were classified as Risk 3 (minor). The compliance rate was 97% [37].

Comparing the results with other HRA, ANVISA had more unsatisfactory results (12.55%) in contrast with the EMA, U.S. FDA, and Health Canada that all found 1–3% of non-compliance results. As shown in Fig 2, this number is higher when the company is inspected for the first time (19.80%), demonstrating that most of previously inspected companies satisfactorily addressed corrective and preventive actions to deficiencies reported and the GMP compliance level has improved.

ANVISA has also been shown to refuse a large number of MA when compared to the U.S. FDA and EMA, with drug product quality control, drug product stability study, active pharmaceutical ingredient quality control made by drug manufacturer, active pharmaceutical ingredient and production report being the main technical reasons for MA refusal in 2015 [38]. These issues are also part of GMP inspections and may reflect on GMP compliance since the most cited deficiency found by ANVISA refers to products not consistently produced and controlled according to the quality standards appropriate to their intended use and as required by the marketing authorization (Art. 13), as noted in Table 4.

Considering the countries that are more frequently inspected by ANVISA, France has the highest non-compliance rate (26.09%) followed by Ireland (25%) and China (20%). From the 40 OAI results reported by the U.S. FDA in the fiscal year 2016, 22 were for U.S. companies, six Chinese, two Indian, and two Brazilian companies [35]. The EMA has issued more non-compliance statements for facilities located in India and China [36]. However, the number of unsatisfactory results obtained by ANVISA in those countries is larger.

Table 2 shows that ANVISA inspects more international manufacturers of nonsterile solids and sterile products. Considering these major groups of pharmaceutical products, sterile products had more non-compliance and on demand results than nonsterile solids, which was to be expected, since sterile products need also comply with additional guidelines (Title III of Brazilian GMP Guidelines). The level of unsatisfactory and on demand results for nonsterile liquids and semisolids was not expected, since the manufacture of these products is considered simpler than solids and sterile products; however, this data may be related to the small number of inspections for these lines.

Considering the frequency of the deficiencies found during ANVISA’s inspections 28.63% of ANVISA-inspected companies in 2015–16 presented some documentation non-compliance item (including records), and 26.27% showed deficiencies related to premises. Regarding the total number of deficiencies, 11.97% were related to premises and 10.15% to documentation. The basic rules of GMP determine that the pharmaceutical manufacturer must maintain proper documentation and records. Documentation ensures traceability of all manufacturing and testing activities, is essential to GMP compliance and allows the auditors to assess the overall quality of operations within a company and the final product [39].

Cooperation and harmonization is an international opportunity for the exchange of knowledge and experiences as well as regulation improvements supporting HRA in their commitment to implement scientific standards in drug registration, inspections, and pharmacovigilance [13]. The Pharmaceutical Inspection Convention/Pharmaceutical Inspection Co-Operation Scheme (PIC/S) has provided an active and constructive forum for cooperation in the field of GMP inspection [40]. The mission of the PIC/S is international development, implementation, and maintenance of harmonized GMP standards, and the quality systems of inspectorates in the field of medicinal products [41]. PIC/S has established a rigorous assessment process for inspectorates and only those meeting the standard are allowed to join the scheme [40]. Currently, 52 HRA are members of PIC/S. Brazil and Armenia are applying for membership. The procedure adopted by ANVISA to classify deficiencies is similar to the SOP established by PIC/S [42] and Health Canada [43], with the exception that PIC/S denominates minor deficiencies as “other.”

In 2011, PIC/S held a workshop on the similarities and differences in the top 10 deficiencies cited by PIC/S members and, before the workshop, a questionnaire was developed and sent to invite all PIC/S members and applicants to share inspection data. The results of this study showed that the most frequently cited categories of GMP deficiencies were “documentation—manufacturing” followed by “design and maintenance of premises” and “documentation—quality systems (elements/procedures)”. The study also showed that there are no significant differences among regions regarding the way GMP deficiencies are identified, classified, and cited, meaning that there is harmonization across PIC/S members. However, different levels of details of the deficiency classes model were noted, and a model was needed for alignment [44].

The U.S. FDA investigators list inspection observations on a form (FDA Form 483). Summaries of the areas of regulation cited and their frequency are available by fiscal year on the U.S. FDA web page [45]. During the fiscal year 2016, the U.S. FDA issued 691 Form 483s for drugs. The most common inspectional observation was “procedures not in writing, fully followed” (147 citations), followed by “scientifically sound laboratory controls” (133 citations) and “investigations of discrepancies, failures” (126 citations).

The most cited item of Brazilian regulation was Article 13, as presented in Table 4, which is related to general GMP requirements. This article summarizes all GMP requirements, establishing that products must be consistently produced and controlled to the quality standards appropriate to their intended use and as required by the MA. Furthermore, it requires that a risk management system is in place to mitigate inherent risks, that instructions and procedures are clear and unambiguous, records are made, and any significant deviations are fully documented and investigated.

The second most cited article was 569; this item, which is related to water systems reviews, describes the topics that must be considered in the examination. Most HRA do not describe this point in such a detailed way in regulation as ANVISA does. It is based on item 7.5 of the World Health Organization (WHO) Technical Report Series 929, 2005—Annex 3 [30], which is a guidance document, supplementary to the general GMP guidelines for pharmaceutical products. Violation of this article is considered a minor deficiency.

Two articles were in the third position of most cited articles with 21 citations in 255 inspections (8.24%). One is related to documentation deficiencies (Article 197), and the other is related to clean and dirty equipment holding times (establishment and validation).

It is necessary that manufacturers are aware of the importance of proper implementation of GMP guidelines and their responsibilities relating to the manufacture of medicinal products, which means an efficient system of quality control and quality risk management [6].

The Brazilian GMP regulation [2] is mostly based on the WHO guidelines. However, some of these guidelines were reviewed by the WHO after the publication of the Brazilian regulations. Additionally, ANVISA recently joined the International Council for Harmonisation of Technical Requirements for Pharmaceuticals for Human Use (ICH) and is applying for PIC/S, meaning that the Brazilian GMP guidelines will need updating in the next few years. PIC/S membership will be valuable for Brazil, because it allows the exchange of GMP information such as inspection reports and recall alerts, aims to harmonize GMP standards and guidance documents, and provides training for competent authorities [46].

The health of people living in developing countries is critically dependent on the availability of good quality medicines [7]. Global and national regulatory systems require significant and urgent reform and strengthening to assure the quality and safety of medicines and contribute to more sustainable health systems and the achievement of universal health coverage [40].

Pharmaceutical manufacture and regulation is a globalized business. Emphasis on harmonization efforts, standard setting, and mutual recognition agreements are increasing. Knowledge of international guidelines is important for understanding the future direction of these efforts [39]. Regulatory convergence, a movement towards technical alignment to enable the adoption of local regulatory mechanisms that consider internationally recognized standards and principles to promote a single sanitary goal, has also emerged to address the challenge of regulating international practices taking into consideration the specific realities of each country [47]. The U.S. FDA created a “risk-based” method for prioritizing GMP inspections [48], developing a model to predict establishments that could have problems in maintaining GMP standards, and inspections are focused on those facilities [49]. In 2012, PIC/S also recommended a model for risk-based inspection planning in the GMP environment [50]. ANVISA adopted PIC/S recommendations in 2014 [51]. However, for international inspections, it is impracticable to cover all the companies that need to be inspected for the maximum time recommended by PIC/S (3 years), as ANVISA receives around 800 applications for the International Medicines GMP certificate per year, and there are more than 1,250 international drug manufacturers registered in the inspection database. Consequently, most of the international GMP certificates are renewed without a new inspection, based on a risk assessment, considering the GMP compliance history, time elapsed since the last inspection, new products or line inclusions, marketing complaints, and product quality review information [24]. HRA should work together for the strengthening of GMP inspections and to avoid duplicated efforts, with the primary objective of assuring medicines comply with the required quality standards. Information sharing within PIC/S is voluntary, and it is up to the receiver of the information to decide how to use it, but a participating authority can use the outcome of an inspection conducted by another PIC/S authority to avoid duplicating an inspection [46]. The Australian Department of Health’s Therapeutic Goods Administration (TGA) is using a GMP clearance system for companies certified by PIC/S members and has significantly reduced the number of overseas required inspections [12]. In this context, becoming a PIC/S member could make the best use of ANVISA’s resources to focus on high-risk facilities in Brazil or overseas.

Disclosure of the leading GMP deficiencies is a key step for regulatory transparency and can be useful for both industry and ANVISA to improve the regulation process. As noted above, different regulatory approaches and practices have been adopted by HRAs for publicizing results of inspections. Presently, ANVISA only publishes the results of certification applications on the Government Official Gazette and in a database available, in Portuguese, on its website [52]. It may be useful to adopt an annual review on common GMP deficiencies, as recommended by PIC/S [44], to identify specific GMP areas to be focused on in training, harmonization and targeting the GMP inspections to high-risk facilities. Furthermore, additional guidelines in the specific areas where deficiencies are often identified may be useful for the industry to improve GMP compliance.

Reported data shows that ANVISA inspections have observed a more substantial number of unsatisfactory (non-compliance) results than other stringent regulatory authorities, which may be explained by the different procedures adopted by each HRA for the conclusion on the status of facilities, as the pattern of deficiencies found is similar to other authorities. Another possible explanation for the obtained results is that manufacturers inspected by ANVISA use different standards and practices for products manufactured to the Brazilian market. Brazil is an attractive market for both innovative and generic medicines because the government covers many types of treatment, and they represent a significant portion of the health budget [53]. Without assurance that these medicines meet acceptable standards of quality, safety, and efficacy, health services may be compromised [54]. International inspections conducted by ANVISA are important to prevent poor quality pharmaceutical products entering the supply chain in Brazil. However, the results obtained reinforce the need for harmonization of inspection procedures and decisions between HRAs to avoid duplicated work and prioritize high-risk facilities with the main objective that patients receive safe, effective and quality medicines.

## Conclusion

The most common areas of deficiencies reported by ANVISA during international GMP inspections of medicines were related to documentation and premises, similar to the findings of stringent regulatory agencies such as the U.S. FDA and PIC/S members, showing that similar inspection procedures are applied. However, considering the compliance rate, ANVISA found a higher number of non-compliance results than other authorities, which may be caused by different types of decision adopted by each jurisdiction or the standards applied for manufacturing products to Brazilian market may not be the same as used in those markets. Our findings emphasize the need to promote the use of medicines manufactured under GMP conditions to prevent substandard medicine circulation, especially in developing countries. ANVISA could conduct this kind of evaluation periodically to identify patterns and trends, focusing on inspections at high-risk manufacturers, as well as detecting divergent issues with international regulations, seeking regulatory convergence and international harmonization of GMP guidelines and inspection procedures. In addition, disclosure of the common deficiencies found contributes to regulatory transparency, which may be useful for the industry to improve GMP compliance. International collaboration on GMP inspections could avoid duplication of efforts between regulatory authorities, allowing them to take a risk-based approach and focus on inspections of non-compliant companies, increasing good quality medicines availability and contributing to a sustainable public health system.

## References

[1] WHO Expert Committee on Specifications for Pharmaceutical Preparations. WHO good manufacturing practices for pharmaceutical products: main principles. WHO Tech Rep Ser. 2014;986: 78–135.

[2] ANVISA. Resolução RDC no 17 de 16 de abril de 2010. Diário Of da União. 2010;19 abr: 94–110.

[3] EMA. EU Guidelines for Good Manufacturing Practice for Medicinal Products for Human and Veterinary Use. EudraLex. 2012;4: 1–8. 10.2903/j.efsa.2015.4206.OJ

[4] PIC/S. Guide To Good Manufacturing Practice for Medicinal Products. 2017;

[5] FDA. Facts About the Current Good Manufacturing Practices (cGMPs) [Internet]. 2015 [cited 28 May 2017] pp. 1–3. Available: http://www.fda.gov/Drugs/DevelopmentApprovalProcess/Manufacturing/ucm169105.htm

[6] Reis C, Gouveia B, Rijo P, Gonçalo T. Good manufacturing practices for medicinal products for human use. J Pharm Bioallied Sci. 2015;7: 87 10.4103/0975-7406.154424 25883511 PMC4399016

[7] Newton PN, Lee SJ, Goodman C, Fernández FM, Yeung S, Phanouvong S, et al Guidelines for field surveys of the quality of medicines: A proposal. PLoS Med. 2009;6: 0252–0257. 10.1371/journal.pmed.1000052 19320538 PMC2659710

[8] Lukulay PH, El-Hadri L, Raymond C, Hajjou M, Roth L, Boateng KP, et al Monitoring the Quality of Medicines: Results from Africa, Asia, and South America. Am J Trop Med Hyg. 2015;92 10.4269/ajtmh.14-0535 25897073 PMC4455073

[9] Johnston A, Holt DW. Substandard drugs: A potential crisis for public health. Br J Clin Pharmacol. 2014;78 10.1111/bcp.12298 24286459 PMC4137817

[10] Senior K. Global health-care implications of substandard medicines. Lancet Infect Dis. 2008;8: 666 10.1016/S1473-3099(08)70241-X 18992395

[11] Newton PN, Amin AA, Bird C, Passmore P, Dukes G, Tomson G, et al The primacy of public health considerations in defining poor quality medicines. PLoS Med. 2011;8: 6–10. 10.1371/journal.pmed.1001139 22162953 PMC3232210

[12] Jin H, Carr N, Rothenfluh H. Regulating medicine manufacturers: is an on-site inspection the only option? WHO Drug Inf. 2017;31: 153–7.

[13] Sousa VD, Ramalho PI, Silveira D. Sharing regulatory data as tools for strengthening health systems in the Region of the Americas. Pan Am J Public Heal. 2016;39: 1–10. 27706398

[14] Woodcock J. Reliable Drug Quality: An Unresolved Problem. PDA J Pharm Sci Tech. 2012;66: 270–272. 10.5731/pdajpst.2012.00868 22634592

[15] Patel DS, Patel AR, Patel NA. The FDA cGMP inspection is coming: make the best of it. J GXP Compliance. 2012;16: 64.

[16] Fox ER, Birt A, James K., Kokko H, Salverson S, Soflin DL. ASHP Guidelines on Managing Drug products in Hospitals and Health Systems. Am J Heal Pharm. 2009;66: 1399–1405. 10.2146/ajhp090026 19635779

[17] Sifferlen SC, Sifferlen SCHA. Drug Shortages, Today and Tomorrow − An Industry Perspective. PDA J Pharm Sci Tech. 2015;69: 557–561. 10.5731/pdajpst.2015.01066 26242791

[18] De Weerdt E, Simoens S, Hombroeckx L, Casteels M, Huys I. Causes of drug shortages in the legal pharmaceutical framework. Regul Toxicol Pharmacol. Elsevier Inc.; 2015;71: 251–258. 10.1016/j.yrtph.2015.01.005 25591547

[19] Li E, Subramanian J, Anderson S, Thomas D, McKinley J, Jacobs IA. Development of biosimilars in an era of oncologic drug shortages. Drug Des Devel Ther. 2015;9: 3247–55. 10.2147/DDDT.S75219 26150698 PMC4484646

[20] Fox ER, Sweet B V., Jensen V. Drug shortages: A complex health care crisis. Mayo Clin Proc. 2014; 10.1016/j.mayocp.2013.11.014 24582195

[21] Mazer-Amirshahi M, Pourmand A, Singer S, Pines JM, Van Den Anker J. Critical drug shortages: Implications for emergency medicine. Acad Emerg Med. 2014;21: 704–711. 10.1111/acem.12389 25039558

[22] ANVISA. About Anvisa [Internet]. [cited 17 Aug 2017]. Available: http://portal.anvisa.gov.br/contact-us

[23] ANVISA. Resolução—RDC no 25, de 9 de dezembro de 1999. Diário Of da União. 1999;10/12/99: 32.

[24] ANVISA. Resolução RDC no 39 de 14 de agosto de 2013. Diário Of da União. 2013;15 ago: 50–52.

[25] SNVS. POP-O SNVS-014-Categorização de não conformidades, classificação de estabelecimentos quanto ao cumprimento das boas práticas e determinação do risco regulatório [Internet]. 2014 [cited 17 Aug 2017]. Available: http://portal.anvisa.gov.br/documents/33864/0/Selecao+de+Procedimentos+SNVS/a9d23041-d676-4109-aec5-5a23fef71c70?version=1.1

[26] WHO Expert Committee on Specifications for Pharmaceutical Preparations. Good Manufacturing Practices for pharmaceutical products: main principles. WHO Tech Rep Ser. 2003;908: 36–89.

[27] WHO Expert Committee on Specifications for Pharmaceutical Preparations. Good manufacturing practices for sterile pharmaceutical products. WHO Tech Rep Ser. 2002;902: 76–93. 12082969

[28] WHO Expert Committee on Specifications for Pharmaceutical Preparations. Good manufacturing practices for biological products. WHO Tech Rep Ser. 1992;822: 20–30.

[29] WHO Expert Committee on Specifications for Pharmaceutical Preparations. Supplementary guidelines on good manufacturing practices: validation. WHO Tech Rep Ser. 2006;937: 107–178.

[30] WHO Expert Committee on Specifications for Pharmaceutical Preparations. WHO Good Manufacturing Practices: water for pharmaceutical use. WHO Tech Rep Ser. 2005;929: 40–58. 16353684

[31] EMA. Good Manufacturing Practice Medicinal Products for Human and Veterinary Use—Annex 11: Computerised Systems. EudraLex. 2011.

[32] PIC/S. PI 011—Good Practices for Computerised Systems in Regulated “Gxp” Environments. 2007; 1–54. doi:PI 011–3

[33] WHO Expert Committee on Specifications for Pharmaceutical Preparations. Supplementary guidelines on good manufacturing practices for the manufacture of herbal medicines. WHO Tech Rep Ser. 2006;937: 85–106.

[34] FDA. Inspections [Internet]. [cited 28 May 2017]. Available: https://www.fda.gov/downloads/aboutfda/transparency/publicdisclosure/glossaryofacronymsandabbreviations/ucm212061.pdf

[35] FDA. Inspection Classification Database Search [Internet]. [cited 17 Aug 2017]. Available: https://www.accessdata.fda.gov/scripts/inspsearch/

[36] EMA. Annual Report 2016 [Internet]. [cited 17 Aug 2017]. doi:10.1017/CBO9781107415324.004

[37] Health Canada. Inspectorate Program Annual Inspection Summary Report 2014–2015 [Internet]. [cited 17 Aug 2017]. Available: http://www.hc-sc.gc.ca/dhp-mps/alt_formats/pdf/pubs/compli-conform/2014-2015-ar-ra/2014-2015-ar-ra-eng.pdf

[38] Carmo ACM do, Piras SS, Rocha NFM, Gratieri T. Main Reasons for Registration Application Refusal of Generic and Similar Pharmaceutical Drug Products by the Brazilian Health Regulatory Agency (ANVISA). In: BioMed Research International 2017 pp. 1–10. 10.1155/2017/7894937 PMC5322421 28280742

[39] Patel KT, Chotai NP. Documentation and Records: Harmonized GMP Requirements. J Young Pharm. 2011;3: 138–50. 10.4103/0975-1483.80303 21731360 PMC3122044

[40] Wirtz VJ, Hogerzeil H V, Gray AL, Bigdeli M, Joncheere CP de, Ewen MA, et al Essential medicines for universal health coverage. Lancet. 2017;389: 403–76. 10.1016/S0140-6736(16)31599-9 27832874 PMC7159295

[41] PIC/S. Mission, Vision and Values [Internet]. [cited 6 Jul 2017]. Available: https://www.picscheme.org/en/mission-vision-and-values

[42] PIC/S. PIC/S Standard Operating Procedure—PIC/S Inspection Report Format [Internet]. 2007 [cited 28 May 2017]. Available: https://www.picscheme.org/layout/document.php?id=137

[43] Health Canada. Risk Classification of Good Manufacturing Practices (GMP) Observations [Internet]. 2012 [cited 28 May 2017]. Available: http://www.hc-sc.gc.ca/dhp-mps/alt_formats/pdf/compli-conform/gmp-bpf/docs/gui-0023-eng.pdf

[44] Smallenbroek H, Hoe BM. Top GMP Deficiencies. Pharm Technol. 2012;36: 135.

[45] FDA. Inspection Observations [Internet]. 2017 [cited 9 Apr 2017]. Available: https://www.fda.gov/iceci/inspections/ucm250720.htm

[46] Gråberg T. Inside PIC/S: The Importance of PIC/S in a Globalized World. Pharm Technol. 2011;35.

[47] Silva APJ, Tagliari POP. Iniciativas de convergência regulatória em saúde nas Américas: histórico, evolução e novos desafios. Pan Am J Public Heal. 2016;39: 281–287. 27706413

[48] FDA. Pharmaceutical cGMPS for the 21st Century—a Risk-Based Approach Final Report [Internet]. 2004 [cited 12 Apr 2017]. Available: https://www.fda.gov/downloads/drugs/developmentapprovalprocess/manufacturing/questionsandanswersoncurrentgoodmanufacturingpracticescgmpfordrugs/ucm176374.pdf

[49] Stuart O. Schweitzer. Trying Times at the FDA—The Challenge of Ensuring the Safety of Imported Pharmaceuticals. N Engl J Med. 2008;358: 1773–1777. 10.1056/NEJMp0802041 18434648

[50] PIC/S. PI 037–1—A Recommended Model for Risk-Based Inspection Planning in the GMP Environment [Internet]. 2012 [cited 28 May 2017]. Available: https://www.picscheme.org/layout/document.php?id=160

[51] SNVS. POP-O-SNVS-015—Planejamento de Inspeções para Verificação das Boas Práticas de Fabricação de Medicamentos e Insumos Farmacêuticos com Base no Risco Sanitário Associado. [Internet]. 2014 [cited 17 Aug 2017]. Available: http://portal.anvisa.gov.br/documents/33864/0/Selecao+de+Procedimentos+SNVS/a9d23041-d676-4109-aec5-5a23fef71c70?version=1.1

[52] ANVISA. Certificado de Boas Práticas [Internet]. [cited 12 Apr 2017]. Available: http://www.anvisa.gov.br/certificadoBoasPraticas/principal/index.asp

[53] Castanheira LG, Barbano DBA, Rech N. Current development in regulation of similar biotherapeutic products in Brazil. Biologicals. Elsevier Ltd; 2011;39: 308–311. 10.1016/j.biologicals.2011.06.021 21868247

[54] WHO. Quality assurance of pharmaceuticals: a compendium of guidelines and related materials. 2nd ed World Health Organization; 2007 10.1097/NCN.0b013e3182148ed0

